# Impact of Postoperative Infectious Complications on Long-Term Outcomes for Patients Undergoing Simultaneous Resection for Colorectal Cancer Liver Metastases: A Propensity Score Matching Analysis

**DOI:** 10.3389/fonc.2021.793653

**Published:** 2022-01-07

**Authors:** Qichen Chen, Yiqiao Deng, Jinghua Chen, Jianjun Zhao, Xinyu Bi, Jianguo Zhou, Zhiyu Li, Zhen Huang, Yefan Zhang, Xiao Chen, Hong Zhao, Jianqiang Cai

**Affiliations:** Department of Hepatobiliary Surgery, National Cancer Center/National Clinical Research Center for Cancer/Cancer Hospital, Chinese Academy of Medical Sciences and Peking Union Medical College, Beijing, China

**Keywords:** colorectal cancer liver metastases, simultaneous resection, propensity score matched, long-term outcomes, postoperation infection

## Abstract

**Objective:**

To investigate the impact of postoperative infectious complications (POI) on the long-term outcomes of patients with colorectal cancer liver metastasis (CRLM) after simultaneous resection of colorectal cancer and liver metastases.

**Methods:**

Four hundred seventy-nine CRLM patients receiving simultaneous resection between February 2010 and February 2018 at our hospital were enrolled. A 1:3 propensity score matching analysis (PSM) analysis was performed to balance covariates and avoid selection bias. After PSM, 90 patients were distributed to the POI group, and 233 patients were distributed to the no POI group. A log-rank test was performed to compare the progression-free survival (PFS) and overall survival (OS) data. A multivariate Cox regression model was employed to identify prognostic factors influencing OS and PFS. A value of two-sided *P*<0.05 was considered statistically significant.

**Results:**

Compared to patients in the no POI group, patients in the POI group were more likely to have hepatic portal occlusion (78.9% vs. 66.3%, *P*=0.021), operation time ≥325 min (61.1% vs. 48.1%, *P*=0.026), and intraoperative blood loss ≥200 ml (81.1% vs. 67.6%, *P*=0.012). In multivariate analysis, intraoperative blood loss ≥200 ml (OR = 2.057, 95% CI: 1.165-3.634, *P*=0.013) was identified as the only independent risk factor for POI. Patients with POI had a worse PFS (*P<*0.001, median PFS: 7.5 vs. 12.7 months) and a worse OS (*P*=0.010, median OS: 38.8 vs. 59.0 months) than those without POI. After 1:3 PSM analysis, no differences in clinicopathologic parameters were detected between the POI group and the no POI group. Patients with POI had a worse PFS (*P*=0.013, median PFS: 7.5 vs. 11.1 months) and a worse OS (*P*=0.020, median OS: 38.8 vs. 59.0 months) than those without POI. Multivariate analysis showed that POI was an independent predictor for worse PFS (HR=1.410, 95% CI: 1.065-1.869, *P*=0.017) and worse OS (HR=1.682, 95% CI: 1.113-2.544, *P*=0.014).

**Conclusions:**

POI can significantly worsen the long-term outcomes of CRLM patients receiving simultaneous resection of colorectal cancer and liver metastases and should be considered to improve postoperative management and make better treatment decisions for these patients.

## Introduction

Colorectal cancer (CRC) is the third most common malignant cancer and the second leading cause of cancer-related death in the world (1). The liver is the most common metastatic site, and more than 50% (2) of patients with colorectal cancer will develop liver metastases during their lifetimes. Liver metastases are often the cause of death for these colorectal cancer liver metastasis (CRLM) patients.

Surgery remains the only curative method for these patients. Traditionally, surgeons usually choose staged resection of the primary lesion and liver metastases in two separate operations (3). However, with the development of surgical technology, the improvement of the safety of hepatectomy (4) and the successful preoperative systematic treatment (5), simultaneous resection of the primary tumor and liver metastases in one operation has been increasing (6). Simultaneous resection has the advantages of reducing medical costs (6) and lowering the risk of other metastatic diseases in the interval of primary tumor resection and liver metastasis resection. In addition, several retrospective studies (7–9) revealed that patients receiving simultaneous resection could have comparable long-term outcomes to those receiving staged resection. Moreover, a recent randomized controlled trial (10) revealed that long-term outcomes tended to be improved in the simultaneous resection group compared with the staged resection group.

Although simultaneous resection has been advocated by clinicians because of the above advantages, the procedure is associated with increased postoperative complications (POCs) (11–14). Postoperative infectious complications (POI), as one of the major POCs, occur in 4-22 (15) percent of patients who undergo surgical resection for malignant solid tumous,and have been proven to have a negative impact on the long-term outcomes for patients with cancers (16–18), including colorectal cancer (19), oesophageal cancer (17) and gastric cancer (16). However, the impact of POI on CRLM patients is not fully understood, and there is a lack of evidence about how POI affects the long-term outcomes of patients receiving simultaneous resection of colorectal cancer and liver metastases. The present study aimed to explore the impact of POI on the long-term outcome of CRLM patients receiving simultaneous resection and identify the predictive factors of POI to help improve postoperative management of these patients.

## Methods

### Data Collection and Patient Population

The study was approved by the Institutional Review Board of the Cancer Hospital, Chinese Academy of Medical Sciences. The inclusion criteria were as follows: (I) pathologically proven liver metastases of colorectal adenocarcinoma and (II) treatment with simultaneous resection of the primary tumor and liver metastases for curative purposes. Patients were excluded for incomplete follow-up data, lack of clinical data or the presence of other malignant tumors. Then, the clinical data of 479 CRLM patients admitted to the hospital between February 2010 and February 2018 were collected and analysed retrospectively.

Detailed information on demographics, clinicopathological characteristics, medical treatment and oncological results was reviewed. Diverse POCs, such as respiratory system infection, urinary system infection, digestive system infection, wound infection, and sepsis, were defined as POI (19). Patients were divided into two groups (POI, n=90; no POI, n=389). Of those POCs, minor complications were defined as Clavien-Dindo I-II, while major complications were defined as Clavien-Dindo III-V *via* the Clavien-Dindo classification system.

### Treatment

Appropriate treatment strategies for CRLM patients were discussed by a multidisciplinary team (MDT) composed of surgeons, oncologists and radiologists. Preoperative chemotherapy regimens, mainly consisting of 5-fluorouracil/capecitabine and oxaliplatin/irinotecan, with or without bevacizumab and cetuximab, were recommended to patients with any high-risk factors for recurrence (20). Liver resections were divided into major and minor resections. Resections of more than two segments were described as major resection and other resections were defined as minor resection (21). The surgical information mainly included surgical margin, extent of liver resection (major liver resection and minor liver resection), interoperative portal blockade, and concomitant RFA.

### Follow-Up and Outcomes

Patients were followed up with regular clinical examinations after surgery: the first follow-up date was one month after surgery, then every 3 months for 5 years, and every 1 year thereafter. The interval from the date of surgery to death or the last follow-up was defined as overall survival (OS). The interval from the date of surgery to progression or the last follow-up was defined as progression-free survival (PFS).

### Statistical Analysis

Continuous variables are presented as medians with interquartile ranges (IQRs), and the Mann-Whitney U test was used for analysis. Chi-square or Fisher’s exact tests were performed to analyse categorical variables. Multivariable logistic regression analysis was employed to explore the relationships between various variables and POI. Propensity score matching (PSM) was conducted to balance covariates and reduce the selection bias between the POI group and the no POI group. The present study used the Kaplan-Meier method to calculate PFS and OS. To statistically compare the PFS and OS data, the log-rank test was performed. A Cox regression model was employed to identify prognostic factors influencing OS and PFS, with results presented as hazard ratios (HRs) with 95% confidence intervals (CIs). Variables with *P*<0.10 in univariable analysis were included in the multivariable analysis. A value of two-sided *P*<0.05 was considered statistically significant. All statistical analyses were performed using SPSS version 22 software (Armonk NY, USA) and R software (http://www.r-project.org).

## Results

### Clinicopathological Characteristics

A total of 479 patients were enrolled, and most were male (65.1%), with a median age of 59.0 years (IQR 52.0–65.0). Comorbidity was observed in 202 (42.2%) patients. These patients had a median operation time of 325.0 min (IQR 260.0-415.0), and 50.5% of them had an operation time of more than 325.0 min. A median intraoperative blood loss of 200.0 ml (IQR 100.0-400.0) was observed in these patients; and 70.1% of patients had an intraoperative blood loss of more than 200.0 ml. Primary tumors located in the colon were observed in 57.6% of the patients, while primary tumors located in the right hemicolon were observed in 20.5% of the patients. The median diameter of the largest lesion was 2.5 (IQR 1.5–4.0) cm, and 43.0% of the patients had a lesion of larger than 3.0 cm. In addition, 57.0% of the patients had more than one liver metastasis, with a median of 2.0 liver metastases (IQR 1.0–4.0). Of these patients, the proportion of T3–T4 stage was 92.3%, and the proportion of positive lymph node metastases was 73.1%. Two hundred and sixty-seven patients (55.7%) received preoperative chemotherapy, while forty-six patients (9.6%) received concomitant RFA. One hundred eighty-four patients (38.4%) had liver metastases with a bilobar distribution. The proportion of POCs was 48.4% (232/479), while 20.7% (99/479) had major complications and 27.8% (133/479) had minor complications. Ninety patients (90/479, 18.8%) had a POI. Overall, the mean length of hospital stay was 10.0 days (IQR: 9.0-13.0), and admission rate to ICU was 7.5% (36/479). POCs rate was 48.43% (232/479). Major complication occurred in 99 (20.66%) patients while minor complication occurred in 133 (27.76%) patients.

There were no significant differences between the POI group and the no POI group in age, sex, body mass index (BMI), comorbidities, preoperative carcinoembryonic antigen (CEA), metastasis diameter and number, tumor differentiation, tumor location, primary tumor T stage or positive lymph node metastases. Compared to patients without POI, patients with POI were more likely to have hepatic portal occlusion (78.9% vs. 66.3%, *P*=0.021), operation time ≥325 min (61.1% vs. 48.1%, *P*=0.026), a longer hospital stay (*P*<0.001, mean 16.0 vs. 10.0 days) and intraoperative blood loss ≥200 ml (81.1% vs. 67.6%, *P*=0.012). Besides After 1:3 PSM, 90 patients were distributed to the POI group, and 233 patients were distributed to the no POI group. No differences in clinicopathologic parameters were detected between the two groups. The detailed clinicopathologic characteristics of the two groups of patients before and after matching are described in **Tables 1** and **2**, respectively.

**Table 1:** Postoperative infectious complications in CRLM patients before PSM (n=479).

Item	Infection (n = 90)	Non-infection (n = 389)	*P*	All patients (n = 479)
Age ≥60 years, n (%)	44 (48.9%)	177 (45.5%)	0.561	221 (46.1%)
Male	58 (64.4%)	254 (65.3%)	0.879	312 (65.1%)
BMI ≥24kg/m^2^, n (%)	36 (40.0%)	191 (49.1%)	0.119	227 (47.4%)
Comorbidity, n (%)	41 (45.6%)	161 (41.4%)	0.471	202 (42.2%)
ASA score 3-4, n (%)	13 (14.4%)	45 (11.6%)	0.451	58 (12.1%)
Preoperative CEA ≥10 ng/ml, n (%)	39 (43.3%)	184 (47.3%)	0.497	223 (46.6%)
Primary site in colon, n (%)	56 (62.2%)	220 (56.6%)	0.327	276 (57.6%)
Right hemicolon, n (%)	21 (23.3%)	77 (19.8%)	0.453	98 (20.5%)
Diameter of liver metastases ≥3 cm, n (%)	45 (50.0%)	161 (41.4%)	0.137	206 (43.0%)
Multiple liver metastases, n (%)	53 (58.9%)	220 (56.6%)	0.687	273 (57.0%)
Bilobar liver distribution	34 (37.8%)	150 (38.6%)	0.891	184 (38.4%)
Poor differentiation, n (%)	32 (35.6%)	125 (32.1%)	0.533	157 (32.8%)
T3-T4 stage, n (%)	83 (92.2%)	359 (92.3%)	0.983	442 (92.3%)
Positive lymph node metastasis, n (%)	63 (70.0%)	287 (73.8%)	0.466	350 (73.1%)
Extrahepatic metastases, n (%)	13 (14.4%)	33 (8.5%)	0.084	46 (9.6%)
Concomitant RFA, n (%)	11 (12.2%)	35 (9.0%)	0.349	46 (9.6%)
R0 resection, n (%)	60 (66.7%)	302 (77.6%)	0.029	362 (75.6%)
Major liver resection, n (%)	50 (55.6%)	176 (45.2%)	0.077	226 (47.2%)
Pretreatment chemotherapy, n (%)	54 (60.0%)	213 (54.8%)	0.367	267 (55.7%)
Hepatic portal occlusion, n (%)	71 (78.9%)	258 (66.3%)	0.021	329 (68.7%)
Operation time, min (median, IQR)	370.00 (298.75-450.00)	320.00 (252.00-405.00)	0.002	325.00 (260.00-415.00)
Operation time ≥325min, n (%)	55 (61.1%)	187 (48.1%)	0.026	242 (50.5%)
Blood loss, ml (median, IQR)	200.00 (200.00-525.00)	200.00 (100.00-400.00)	0.008	200.00 (100.00-400.00)
Blood loss ≥200ml, n (%)	73 (81.1%)	263 (67.6%)	0.012	336 (70.1%)
Blood transfusion, n (%)	25 (27.8%)	88 (22.6%)	0.299	113 (23.6%)
The length of hospital stay, day (median, IQR)	16.00 (12.00-22.00)	10.00 (8.00-12.00)	<0.001	10.00 (9.00-13.00)
ICU rate, n (%)	9 (10%)	27 (6.9%)	0.321	36 (7.5%)

**Table 2:** Postoperative infectious complications in CRLM patients after PSM (n=323).

Item	Infection (n = 90)	Non-infection (n = 233)	*P*	All patients (n = 323)
Age ≥60 years, n (%)	44 (48.9%)	99 (42.5%)	0.299	143 (44.3%)
Male	58 (64.4%)	158 (67.8%)	0.564	216 (66.9%)
BMI ≥24kg/m^2^, n (%)	36 (40.0%)	115 (49.4%)	0.131	151 (46.7%)
Comorbidity, n (%)	41 (45.6%)	101 (43.3%)	0.720	142 (44.0%)
ASA score 3-4, n (%)	13 (14.4%)	25 (10.7%)	0.353	38 (11.8%)
Preoperative CEA ≥10 ng/ml, n (%)	39 (43.3%)	109 (46.8%)	0.577	148 (45.8%)
Primary site in colon, n (%)	56 (62.2%)	128 (54.9%)	0.236	184 (57.0%)
Right hemicolon, n (%)	21 (23.3%)	41 (17.6%)	0.241	62 (19.2%)
Diameter of liver metastases ≥3 cm, n (%)	45 (50.0%)	107 (45.9%)	0.510	152 (47.1%)
Multiple liver metastases, n (%)	53 (58.9%)	140 (60.1%)	0.844	193 (59.8%)
Bilobar liver distribution	34 (37.8%)	106 (45.5%)	0.210	140 (43.3%)
Poor differentiation, n (%)	32 (35.6%)	76 (32.6%)	0.616	108 (33.4%)
T3-T4 stage, n (%)	83 (92.2%)	222 (95.3%)	0.283	305 (94.4%)
Positive lymph node metastasis, n (%)	63 (70.0%)	172 (73.8%)	0.489	235 (72.8%)
Extrahepatic metastases, n (%)	13 (14.4%)	26 (11.2%)	0.416	39 (12.1%)
Concomitant RFA, n (%)	11 (12.2%)	19 (8.2%)	0.259	30 (9.3%)
R0 resection, n (%)	60 (66.7%)	166 (71.2%)	0.421	226 (70.0%)
Major liver resection, n (%)	50 (55.6%)	120 (51.5%)	0.513	170 (52.6%)
Pretreatment chemotherapy, n (%)	54 (60.0%)	129 (55.4%)	0.451	183 (56.7%)
Hepatic portal occlusion, n (%)	71 (78.9%)	183 (78.5%)	0.945	254 (78.6%)
Operation time ≥325min, n (%)	55 (61.1%)	136 (58.4%)	0.653	191 (59.1%)
Blood loss, ml (median, IQR)	200.00 (200.00-525.00)	200.00 (200.00-500.00)	0.541	200.00 (200.00-500.00)
Blood loss ≥200ml, n (%)	73 (81.1%)	183 (78.5%)	0.610	256 (79.3%)
Blood transfusion, n (%)	25 (27.8%)	65 (27.8%)	0.983	90 (27.9%)

### Predictors for POI

Among the patients, the proportion of patients with POI was 18.8% (90/479). In the univariate analysis (**Table 3**), operation time ≥325 min (*P*=0.027), intraoperative blood loss ≥200 mL (*P*=0.013) and hepatic portal occlusion (*P*=0.022) were significantly associated with POI. In addition, extrahepatic metastases (*P*=0.087) and major liver resection (*P*=0.079) showed a tendency towards POI. A multivariable logistic regression analysis was performed to identify factors that were independently associated with POI. The above predictors (*P*<0.10) were included in the multivariate analysis, and intraoperative blood loss ≥200 ml (OR = 2.057, 95% CI: 1.165-3.634, *P*=0.013) was identified as an independent predictive factor of POI (**Table 3**).

**Table 3:** Prognostic factors for postoperative infectious complications in CRLM patients before PSM.

Factor	Univariate analysis		Multivariate analysis	
	*P*	OR (95%CI)	*P*	OR (95%CI)
Age ≥60 years	0.561	1.146 (0.724-1.813)		
Male	0.879	0.963 (0.596-1.556)		
BMI ≥24kg/m^2^	0.120	0.691 (0.434-1.102)		
Comorbidity	0.471	1.185 (0.747-1.879)		
ASA score 3-4	0.452	1.291 (0.664-2.509)		
CEA ≥10 ng/ml	0.497	0.852 (0.537-1.352)		
Primary site in colon	0.328	1.265 (0.790-2.026)		
Right hemicolon	0.454	1.233 (0.713-2.134)		
Diameter of liver metastases ≥3 cm	0.138	1.416 (0.894-2.243)		
Multiple liver metastases	0.687	1.100 (0.691-1.752)		
Bilobar liver distribution	0.891	0.967 (0.603-1.552)		
T3-T4 stage	0.983	0.991 (0.421-2.334)		
Positive lymph node metastasis	0.467	0.829 (0.501-1.373)		
Extrahepatic metastases	0.087	1.821 (0.916-3.622)		
Concomitant RFA	0.351	1.408 (0.685-2.893)		
Major liver resection	0.079	1.513 (0.954-2.399)		
Pretreatment chemotherapy	0.367	1.239 (0.777-1.976)		
Hepatic portal occlusion, n (%)	0.022	1.897 (1.097-3.283)		
Operation time ≥325min	0.027	1.697 (1.063-2.711)		
Intraoperative blood loss ≥200ml	0.013	2.057 (1.165-3.634)	0.013	2.057 (1.165-3.634)
Blood transfusion	0.300	1.316 (0.783-2.210)		

### Impact of POI on Long-Term Outcomes Before PSM

**Figure 1:**
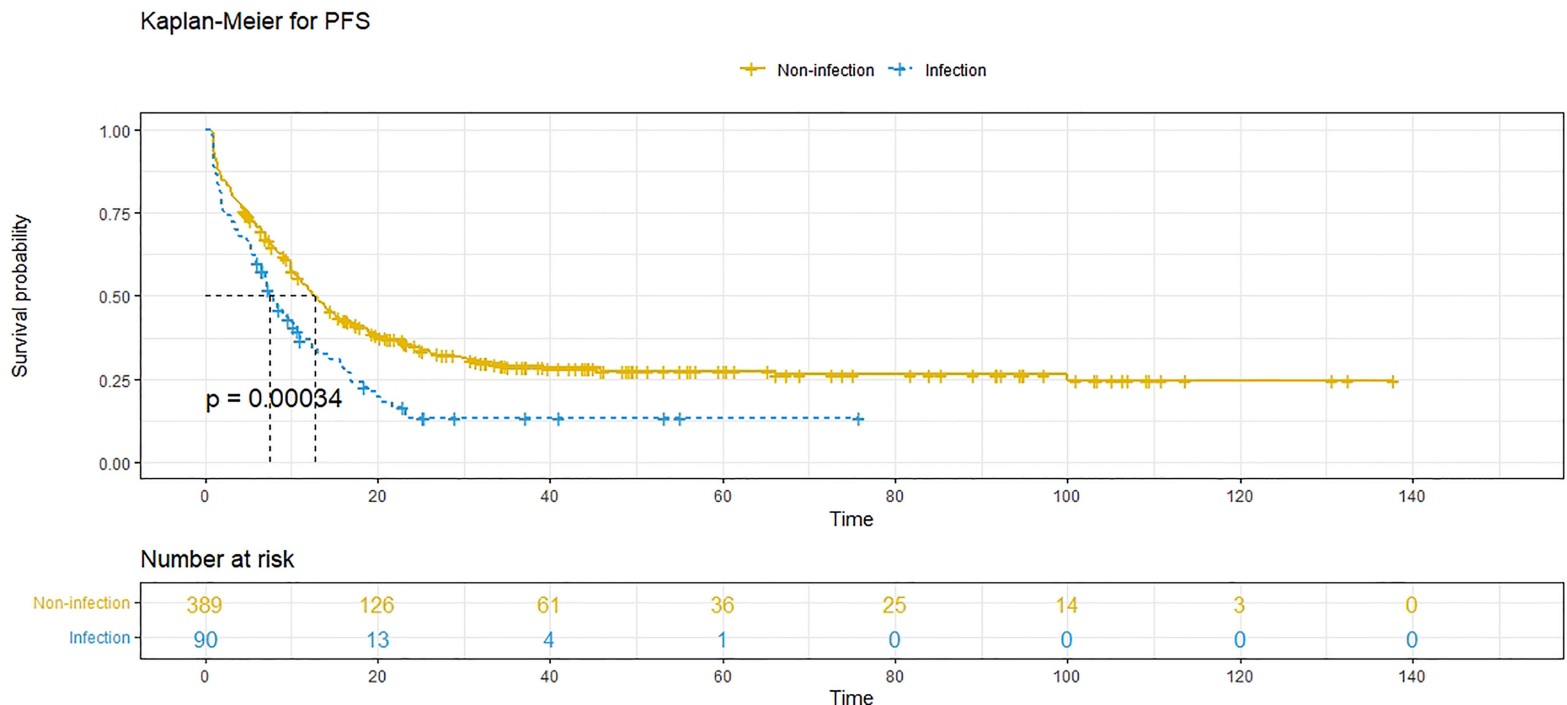
Progression-free survival (PFS) comparison in patients with postoperative infective complications (blue line) and patients without postoperative infective complications (yellow line). Infection, postoperative infective complications. Non-infection, without postoperative infective complications.

**Figure 2:**
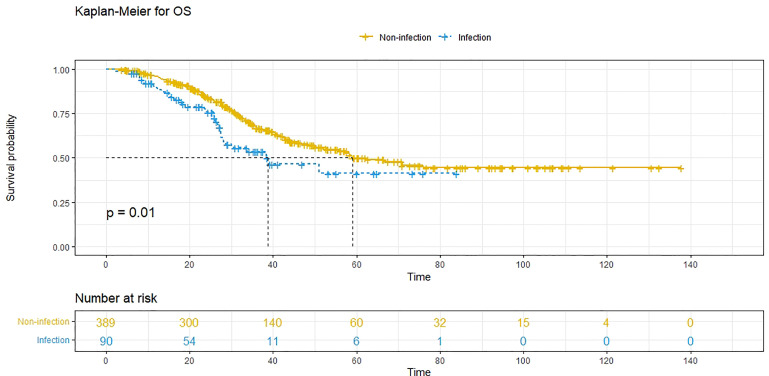
Overall survival (OS) comparison in patients with postoperative infective complications (blue line) and patients without postoperative infective complications (yellow line). Infection, with postoperative infective complication. Non-infection, without postoperative infective complication.

At the time of analysis, 171 patients (35.7%) had died, and 333 patients (69.5%) had experienced recurrence. The median PFS was 11.7 (95% CI: 10.3-13.1) months, and the 1-year, 3-year and 5-year PFS rates were 48.8%, 26.0% and 24.7%, respectively. The median OS was 58.3 (95% CI: 45.4-71.1) months, and the 1-year, 3-year and 5-year survival rates were 94.7%, 64.4% and 48.2%, respectively. Compared to patients without POI, patients with POI had a worse PFS (*P*<0.001, median PFS: 7.5 vs. 12.7 months) (**Figure 1**) and a worse OS (*P*=0.010, median OS: 38.8 vs. 59.0 months) (**Figure 2**).

Univariate analysis showed that a diameter of liver metastases ≥3 cm, multiple liver metastases, bilobar liver distribution, poor differentiation, positive lymph node metastases, extrahepatic metastases, concomitant RFA, non-R0 resection, major liver resection, hepatic portal occlusion, operation time ≥325 min and POI were relevant (*P*<0.05) to a decreased PFS, while intraoperative blood loss ≥200 ml had a tendency (*P*<0.10) towards a decreased PFS. Six independent prognostic factors for PFS were identified in the multivariable analysis: positive lymph node metastases (HR=1.910, 95% CI: 1.453-2.511, *P*<0.001), extrahepatic metastases (HR=1.783, 95% CI: 1.273-2.449, *P*=0.001), R0 resection (HR=0.643, 95% CI: 0.505-0.819, *P*<0.001), major liver resection (HR=1.525, 95% CI: 1.210-1.920, *P*<0.001), operation time ≥325 min (HR=1.349, 95% CI: 1.072-1.697, *P=*0.011), and POI (HR=1.451, 95% CI: 1.110-1.896, *P=*0.006) (**Table 4**).

**Table 4:** Prognostic factors for PFS in CRLM patients before PSM.

Factor	Univariate analysis		Multivariate analysis	
	*P*	HR (95%CI)	*P*	HR (95%CI)
Age ≥60 years	0.983	0.998 (0.804-1.238)		
Male	0.575	1.067 (0.852-1.336)		
BMI≥24kg/m^2^	0.343	1.110 (0.895-1.376)		
Comorbidity	0.916	1.012 (0.814-1.258)		
ASA score 3-4	0.670	0.931 (0.669-1.295)		
CEA≥10 ng/ml	0.937	1.009 (0.813-1.251)		
Primary site in colon	0.607	0.945 (0.761-1.173)		
Right hemicolon	0.976	0.996 (0.761-1.304)		
Diameter of liver metastases ≥3 cm	0.016	1.303 (1.050-1.617)		
Multiple liver metastases	<0.001	1.747 (1.396-2.186)		
Bilobar liver distribution	<0.001	1.796 (1.445-2.232)		
Poor differentiation	0.035	1.279 (1.018-1.607)		
T3-T4 stage	0.113	1.429 (0.919-2.224)		
Positive lymph node metastasis	<0.001	2.058 (1.572-2.695)	<0.001	1.910 (1.453-2.511)
Extrahepatic metastases	<0.001	2.064 (1.476-2.886)	0.001	1.783 (1.273-2.449)
Concomitant RFA	0.001	1.725 (1.234-2.413)		
R0 resection	<0.001	0.552 (0.435-0.701)	<0.001	0.643 (0.505-0.819)
Major liver resection	<0.001	1.870 (1.504-2.325)	<0.001	1.525 (1.210-1.920)
Hepatic portal occlusion, n (%)	0.025	1.308 (1.035-1.654)		
Operation time ≥325 min	<0.001	1.572 (1.265-1.954)	0.011	1.349 (1.072-1.697)
Intraoperative blood loss ≥200 ml	0.058	1.263 (0.992-1.608)		
Blood transfusion	0.361	1.122 (0.876-1.437)		
Pretreatment chemotherapy	0.271	1.130 (0.909-1.404)		
Postoperative infectious complications	<0.001	1.609 (1.235-2.096)	0.006	1.451 (1.110-1.896)

Univariate analysis revealed that a diameter of liver metastases ≥3 cm, multiple liver metastases, bilobar liver distribution, T3-T4 stage, positive lymph node metastases, concomitant RFA, non-R0 resection, major liver resection, hepatic portal occlusion, operation time ≥325 min, blood transfusion, pretreatment chemotherapy and POI were associated (*P*<0.05) with decreased OS. Multivariate analysis revealed that T3-T4 stage (HR=3.228, 95% CI: 1.324-7.865, *P*=0.010), positive lymph node metastases (HR=2.676, 95% CI: 1.741-4.113, *P*<0.001), concomitant RFA (HR=1.678, 95% CI: 1.109-2.538, *P*=0.014), major liver resection (HR=1.427, 95% CI: 1.029-1.979, *P*=0.033), and operation time ≥325 min (HR=1.735, 95% CI: 1.254-2.401, *P*=0.001) were independent prognostic predictors of OS. However, POI (HR=1.468, 95% CI: 1.000-2.155, *P*=0.050) was not independently associated with OS (**Table 5**).

**Table 5:** Prognostic factors for OS in CRLM patients before PSM.

Factor	Univariate analysis		Multivariate analysis	
	*P*	HR (95%CI)	*P*	HR (95%CI)
Age ≥60 years	0.219	1.207 (0.894-1.631)		
Male	0.476	0.894 (0.656-1.217)		
BMI ≥24kg/m^2^	0.701	1.061 (0.786-1.432)		
Comorbidity	0.776	0.957 (0.706-1.297)		
ASA score 3-4	0.403	0.816 (0.506-1.315)		
CEA ≥10 ng/ml	0.140	1.254 (0.929-1.693)		
Primary site in colon	0.884	0.978 (0.723-1.323)		
Right hemicolon	0.571	1.112 (0.770-1.607)		
Diameter of liver metastases ≥3 cm	0.030	1.396 (1.033-1.887)		
Multiple liver metastases	0.001	1.748 (1.275-2.397)		
Bilobar liver distribution	0.002	1.609 (1.188-2.180)		
Poor differentiation	0.110	1.305 (0.942-1.808)		
T3-T4 stage	0.010	3.221 (1.323-7.843)	0.010	3.228 (1.324-7.865)
Positive lymph node metastasis	<0.001	2.684 (1.755-4.104)	<0.001	2.676 (1.741-4.113)
Extrahepatic metastases	0.292	1.301 (0.797-2.122)		
Concomitant RFA	<0.001	2.108 (1.420-3.129)	0.014	1.678 (1.109-2.538)
R0 resection	<0.001	0.548 (0.399-0.752)		
Major liver resection	<0.001	1.997 (1.472-2.708)	0.033	1.427 (1.029-1.979)
Pretreatment chemotherapy	0.006	1.550 (1.136-2.116)		
Hepatic portal occlusion, n (%)	0.013	1.511 (1.091-2.093)		
Operation time ≥325 min	<0.001	1.935 (1.423-2.631)	0.001	1.735 (1.254-2.401)
Intraoperative blood loss ≥200 ml	0.156	1.271 (0.913-1.769)		
Blood transfusion	0.014	1.515 (1.087-2.111)		
Postoperative infectious complications	0.011	1.635 (1.120-2.388)	0.050	1.468 (1.000-2.155)

### Impact of POI on Long-Term Outcomes After PSM

**Figure 3:**
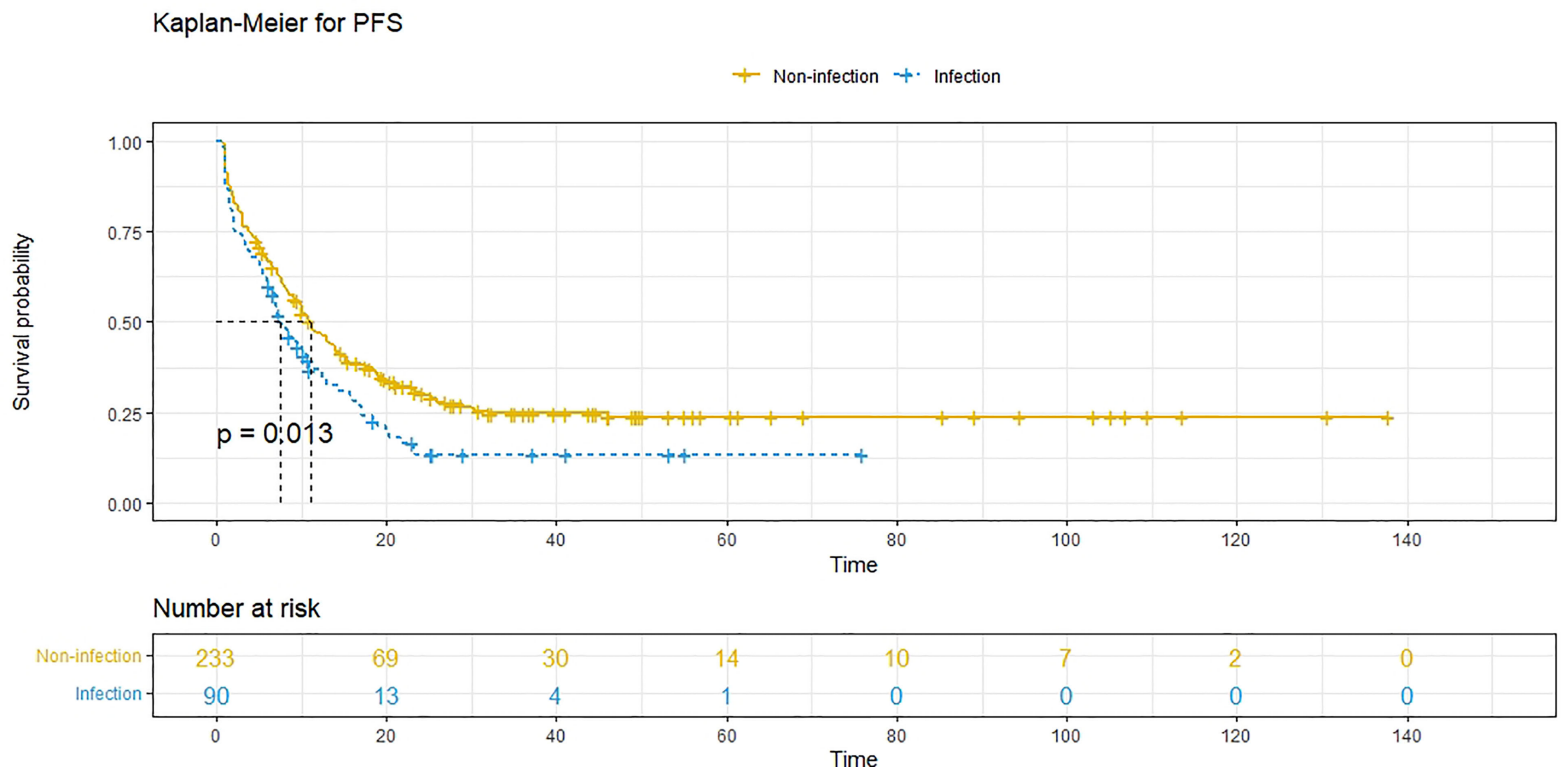
Progression-free survival (PFS) comparison after propensity score matching in patients with postoperative infectious complications (blue line) and patients without postoperative complications (yellow line). Infection, with postoperative infective complication. Non-infection, without postoperative infective complication.

**Figure 4:**
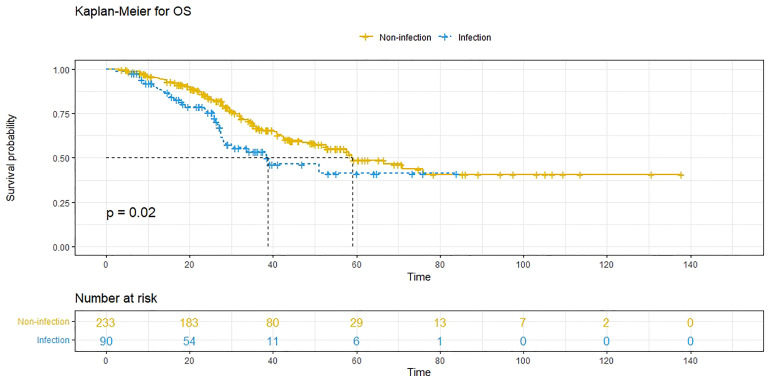
Overall survival (OS) comparison after propensity score matching in patients with postoperative infectious complications (blue line) and patients without postoperative complications (yellow line). Infection, with postoperative infective complication. Non-infection, without postoperative infective complication.

A 1:3 PSM analysis was performed to balance covariates and avoid the selection bias of the retrospective study. Compared to patients without POI, patients with POI had a worse PFS (*P*=0.013, median PFS: 7.5 vs. 11.1 months) (**Figure 3**) and a worse OS (*P*=0.020, median OS: 38.8 vs. 59.0 months) (**Figure 4**). Multivariate analysis showed that POI was an independent predictor of both worse PFS (HR=1.410, 95% CI: 1.065-1.869, *P*=0.017) (**Table 6**) and worse OS (HR=1.682, 95% CI: 1.113-2.544, *P*=0.014) (**Table 7**).

**Table 6:** Prognostic factors for PFS in CRLM patients after PSM.

Factor	Univariate analysis		Multivariate analysis	
	*P*	OR (95%CI)	*P*	OR (95%CI)
Age ≥60 years	0.783	0.965 (0.746-1.247)		
Male	0.511	1.095 (0.836-1.434)		
BMI ≥24kg/m^2^	0.366	1.125 (0.872-1.451)		
Comorbidity	0.848	0.975 (0.754-1.261)		
ASA score 3-4	0.782	0.945 (0.633-1.411)		
CEA ≥10 ng/ml	0.373	1.123 (0.870-1.448)		
Primary site in colon	0.712	0.953 (0.737-1.231)		
Right hemicolon	0.716	1.061 (0.771-1.460)		
Diameter of liver metastases ≥3 cm	0.014	1.374 (1.065-1.773)		
Multiple liver metastases	0.002	1.534 (1.176-2.002)		
Bilobar liver distribution	<0.001	1.575 (1.220-2.032)		
Poor differentiation	0.005	1.464 (1.121-1.911)		
T3-T4 stage	0.021	2.289 (1.131-4.633)	0.030	2.204 (1.081-4.492)
Positive lymph node metastasis	<0.001	2.034 (1.480-2.796)	0.001	1.740 (1.257-2.410)
Extrahepatic metastases	0.004	1.711 (1.185-2.471)	0.029	1.512 (1.044-2.189)
Concomitant RFA	0.047	1.525 (1.006-2.311)		
R0 resection	<0.001	0.576 (0.439-0.755)	0.001	0.633 (0.481-0.834)
Major liver resection	<0.001	1.714 (1.322-2.221)	<0.001	1.676 (1.290-2.178)
Pretreatment chemotherapy	0.670	1.057 (0.818-1.366)		
Hepatic portal occlusion, n (%)	0.539	1.100 (0.812-1.491)		
Operation time ≥325min	0.026	1.345 (1.035-1.747)		
Intraoperative blood loss ≥200ml	0.027	1.459 (1.045-2.036)		
Blood transfusion	0.541	1.091 (0.826-1.440)		
Postoperative infectious complications	0.014	1.420 (1.074-1.879)	0.017	1.410 (1.065-1.869)

**Table 7:** Prognostic factors for OS in CRLM patients after PSM.

Factor	Univariate analysis		Multivariate analysis	
	*P*	HR (95%CI)	*P*	HR (95%CI)
Age ≥60 years	0.477	1.143 (0.791-1.652)		
Male	0.181	0.774 (0.532-1.126)		
BMI ≥24kg/m^2^	0.713	1.071 (0.743-1.544)		
Comorbidity	0.610	0.908 (0.626-1.316)		
ASA score 3-4	0.967	1.012 (0.568-1.804)		
CEA ≥10 ng/ml	0.417	1.164 (0.807-1.678)		
Primary site in colon	0.880	0.972 (0.673-1.405)		
Right hemicolon	0.439	1.188 (0.767-1.840)		
Diameter of liver metastases ≥3 cm	0.049	1.447 (1.002-2.090)		
Multiple liver metastases	0.006	1.739 (1.171-2.582)		
Bilobar liver distribution	0.028	1.511 (1.046-2.182)		
Poor differentiation	0.464	1.161 (0.779-1.730)		
T3-T4 stage	0.061	22.416 (0.871-576.886)		
Positive lymph node metastasis	<0.001	2.963 (1.721-5.101)	<0.001	3.002 (1.736-5.190)
Extrahepatic metastases	0.604	1.160 (0.662-2.033)		
Concomitant RFA	0.004	2.060 (1.258-3.372)	0.058	1.643 (0.983-2.749)
R0 resection	0.003	0.567 (0.390-0.824)		
Major liver resection	0.001	1.867 (1.282-2.719)		
Pretreatment chemotherapy	0.003	1.780 (1.209-2.622)	0.029	1.593 (1.049-2.417)
Hepatic portal occlusion, n (%)	0.085	1.466 (0.949-2.264)		
Operation time ≥325 min	0.002	1.816 (1.234-2.673)	0.029	1.587 (1.049-2.402)
Intraoperative blood loss ≥200 ml	0.168	1.396 (0.869-2.243)		
Blood transfusion	0.014	1.629 (1.105-2.401)		
Postoperative infectious complications	0.021	1.608 (1.073-2.408)	0.014	1.682 (1.113-2.544)

## Discussion

Cancer patients are susceptible to infections, and infection is a significant cause of death in this population (22). For colorectal cancer, bacterial infection (23–25) is of great significance to disease progression, such as metastases. Recently, a study suggested that infection caused by E. coli (26) could contribute to the formation of a protumorigenic environment in the liver and recruit circulating tumor cells, thus promoting liver metastases of colorectal cancer. All these findings indicate that the study of the relationship between infection and CRLM is important for clinical (27) purposes. The present study retrospectively analysed 479 CRLM patients receiving simultaneous resection of colorectal cancer and liver metastases, and PSM was performed to balance the effects of confounding factors. The results revealed that compared with patients in the no POI group, patients in the POI group had a worse PFS and a worse OS before and after PSM. In addition, POI was an independent predictive factor for worse PFS and worse OS in multivariate analysis after PSM. To the best of our knowledge, this is the first study to examine the effect of POI on the long-term outcomes of CRLM patients receiving simultaneous resection of colorectal cancer and liver metastases. Significantly, it can help health care providers improve postoperative management and make better treatment decisions for these patients.

As one of the major POCs, POI remains a concern even in minimally invasive surgeries despite the development of modern surgical technology. Generally, POI not only contributed to longer hospital stays and higher medical costs but was also associated with poor long-term outcomes of patients after surgery for various cancers. Nevertheless, only a few studies have focused on CRLM patients, and previous research is mainly limited to the effect of POI on CRLM patients who receive hepatectomy (28, 29). Regardless of the severity (28), POI was proven to be associated with decreased OS and PFS in CRLM patients who underwent hepatectomy. Compared with previous studies, the present study revealed for the first time that POI was significantly associated with worse PFS and OS in CRLM patients who underwent simultaneous resection.

With regard to the mechanism of the present study, the potential explanation can be postulated as follows: the explanations of previous studies mainly focused on the local or systemic inflammation caused by POI, which can suppress host immunity and promote the proliferation and migration of cancer cells or in the case of bacterial antigen-mediated processes (30–32). Cancer cells can, at the early stage of infection, enhance their metastatic capability by activating Toll-like receptors (TLRs) and creating a prometastatic environment throughout the body (33). Through systemic inflammation, POI can activate micrometastases (34), which cannot be detected by routine postoperative examination, to promote tumor recurrence or progression. More recently, Michela Perego (35) et al. found that stress-induced oxidized lipids can upregulate the fibroblast growth factor pathway in tumor cells, drive the reaction of dormant tumor cells and promote the development of new tumor lesions. Another potential explanation is the postponed postoperative chemotherapy (36, 37), which can be ascribed to POI. The underlying mechanism remains to be fully elucidated, but the clinical utility of our study is still pronounced.

POI occurred in 18.8% (90/479) of patients in the present study, and the incidence was consistent with previous studies (28, 29) of patients who underwent abdominal surgeries. Because of its controllable and preventable nature, it is of great importance to identify the risk factors. In this study, the correlation of various characteristics and POI was analysed by regression analysis. Multivariate analysis indicated that intraoperative blood loss≥200 ml (*P*=0.013) was significantly correlated with POI. The possible explanations of POI caused by intraoperative blood loss are as follows: first, by increasing anti-inflammatory cytokines and prostaglandins, intraoperative blood loss can cause cell-mediated immunosuppression (38); second, intraoperative blood loss can cause damage to the function of the intestinal barrier or break it, which would finally lead to invasion and dislocation of intestinal bacteria (39); third, through the alteration of the immune system, intraoperative blood loss can also increase the relative abundance of opportunistic pathogenic bacterial species in the intestinal tract (40). Given the relationship between intraoperative blood loss and POI, surgeons should take measures to reduce blood loss, such as identifying and managing patients at risk of high blood loss and improving techniques (carefully separating blood vessels, avoiding bleeding in a timely manner, hepatic portal occlusion et al.) to control bleeding during surgery (41). Surgeons can also improve perioperative care, such as maintaining the temperature in the operating room. Su SF et al. (42) found that heating, such as using a forced air heating system to maintain intraoperative normal temperature, can reduce the intraoperative blood loss of patients undergoing surgery. Pu Y et al. (43) also found that the use of a bottom heating system can reduce intraoperative hypothermia in patients undergoing laparoscopic gastrointestinal surgery, thereby reducing intraoperative blood loss. Interestingly, Zei W. et al. (44) reported that gastric cancer patients who underwent neoadjuvant chemotherapy were associated with a higher incidence of POI. However, in the present study, neoadjuvant chemotherapy did not differ in the two groups. It’s worth noting that the impact of neoadjuvant chemotherapy on POI is still controversial, many research reported that for local advanced colorectal cancer (45) and colorectal cancer with liver metastases (46, 47), neoadjuvant chemotherapy would not increase the incidence of postoperative complications including POI. Besides, there were only 90 patients included and patients who underwent combined multiple organ resection were excluded in Zei W.’s study. While in the present study, all 479 CRLM patient underwent simultaneous resection of primary colorectal cancer and liver metastases. The heterogeneity of different cancer and the differences of surgical technique can definitely interfere the results.

There are several limitations in the present study. First, due to the retrospective and single-centre nature of this study, there could be some selection bias because of the lack of necessary randomized grouping. Second, the loss of the number of cases due to PSM may result in the loss of some patient information, and the probability of making the second type of error might increase. In the future, we will expand the sample size and look forward to conducting large prospective studies to further confirm our findings.

In conclusion, this is the first study on the impact of POI on the long-term outcomes of CRLM patients receiving simultaneous resection of colorectal cancer and liver metastases, which provides new decision-making evidence for clinicians to improve the preoperative, intraoperative and postoperative management of these patients. In the future, further application of these findings will help to improve the long-term outcomes of CRLM patients.

## Data Availability Statement

The raw data supporting the conclusions of this article will be made available by the authors, without undue reservation.

## Ethics Statement

The studies involving human participants were reviewed and approved by The Institutional Review Board of the Cancer Hospital, Chinese Academy of Medical Sciences. The patients/participants provided their written informed consent to participate in this study.

## Author Contributions

Conception and design: HZ, JCa. Administrative support: HZ, JCa. Provision of study materials of patients: QC, YD. Collection and assembly of data: All authors. Data analysis and interpretation: QC, YD. Manuscript writing: All authors. Final approval of manuscript: All authors.

## Funding

This study was supported by the National Natural Science Foundation of China (81972311, 82002611), the CAMS Innovation Fund for Medical Sciences (CIFMS) (Grant No. 2017-12M-4-002), the Non-profit Central Research Institution Fund of Chinese Academy of Medical Sciences (2019PT310026) and Sanming Project of Medicine in Shenzhen (No. SZSM202011010).

## Conflict of Interest

The authors declare that the research was conducted in the absence of any commercial or financial relationships that could be construed as a potential conflict of interest.

## Publisher’s Note

All claims expressed in this article are solely those of the authors and do not necessarily represent those of their affiliated organizations, or those of the publisher, the editors and the reviewers. Any product that may be evaluated in this article, or claim that may be made by its manufacturer, is not guaranteed or endorsed by the publisher.
